# Adsorptive removal of acid red 18 dye from aqueous solution using hexadecyl-trimethyl ammonium chloride modified nano-pumice

**DOI:** 10.1038/s41598-023-41100-w

**Published:** 2023-08-24

**Authors:** Mahboobeh Kasraee, Mohammad Hadi Dehghani, Farshad Hamidi, Nabisab Mujawar Mubarak, Rama Rao Karri, Natarajan Rajamohan, Nadeem Hussain Solangi

**Affiliations:** 1 Department of Environmental Health Engineering, School of Public Health, Tehran University of Medical Sciences, Tehran, Iran; 2 Center for Solid Waste Research, Institute for Environmental Research, Tehran University of Medical Sciences, Tehran, Iran; 3 Petroleum and Chemical Engineering, Faculty of Engineering, Universiti Teknologi Brunei, Bandar Seri Begawan, BE1410 Brunei Darussalam; 4 Department of Biosciences, Saveetha School of Engineering, Saveetha Institute of Medical and Technical Sciences, Chennai, India; 5 Chemical Engineering Section, Sohar University, Sohar, Oman; 6 Department of Chemical Engineering, Dawood University of Engineering and Technology, Karachi, 74800 Pakistan

**Keywords:** Environmental sciences, Engineering

## Abstract

Discharging untreated dye-containing wastewater gives rise to environmental pollution. The present study investigated the removal efficiency and adsorption mechanism of Acid Red 18 (AR18) utilizing hexadecyl-trimethyl ammonium chloride (HDTMA.Cl) modified Nano-pumice (HMNP), which is a novel adsorbent for AR18 removal. The HDTMA.Cl is characterized by XRD, XRF, FESEM, TEM, BET and FTIR analysis. pH, contact time, initial concentration of dye and adsorbent dose were the four different parameters for investigating their effects on the adsorption process. Response surface methodology-central composite design was used to model and improve the study to reduce expenses and the number of experiments. According to the findings, at the ideal conditions (pH = 4.5, sorbent dosage = 2.375 g/l, AR18 concentration = 25 mg/l, and contact time = 70 min), the maximum removal effectiveness was 99%. The Langmuir (R^2^ = 0.996) and pseudo-second-order (R^2^ = 0.999) models were obeyed by the adsorption isotherm and kinetic, respectively. The nature of HMNP was discovered to be spontaneous, and thermodynamic investigations revealed that the AR18 adsorption process is endothermic. By tracking the adsorption capacity of the adsorbent for five cycles under ideal conditions, the reusability of HMNP was examined, which showed a reduction in HMNP's adsorption effectiveness from 99 to 85% after five consecutive recycles.

## Introduction

Today’s unstoppable industrial growth will inevitably lead to various environmental issues due to the chemical compounds they use^1–6^. Synthetic dyes are among these compounds, one of the most important industrial substances^6,7^. Azo dyes are regarded as the primary class of synthetic dyes (60–70%) and are extensively employed in a variety of industries, including textile, food, rubber, plastic, paper, and cosmetics Azo dyes are regarded as the primary class of synthetic dyes (60–70%) and are extensively employed in a variety of industries, including textile, food, rubber, plastic, paper, and cosmetics^7–9^. These dyes are formed by an azo group (–N=N–)^8^, low cost, highly stable and soluble^9^. Discharging untreated dye-containing wastewater gives rise to environmental pollution, leading to photosynthesis disturbance by preventing sunlight penetration^10^. Several biological processes can be easily stopped with the presence of dyes in water^11^. It is essential to highlight that ingesting these dyes results in cardiovascular shock, cancer, mutagenesis, teratogenesis, vomiting, gastrointestinal discomfort, diarrhoea, etc^12^.

From this fact, it can be apparent that dye-containing wastewater treatment is a major challenge. Many researchers have investigated several physical, chemical and biological methods for treating coloured wastewater, such as membrane filtration, advanced oxidation techniques, ion exchange, chemical precipitation, coagulation, and flotation^13,14^. However, many of these procedures are unreliable since they fail to remove the colour sufficiently. They are ineffective for dye removal due to their high investment costs, lack of selectivity, and difficulty in regeneration^14,15^.

Adsorption has proven to be an effective technique compared to other methods due to its simplicity of use, high efficiency, and low energy need technology^16,17^. Researchers have recently evaluated pumice stones as a cost-effective adsorbent in water and wastewater purification procedures^17,18^. Pumice is a volcanic, light, porous and nontoxic stone^19^. Open channels inside its structure permit ions and water to enter and exit the crystal lattice^20^. It is a valuable scouring, scrubbing, and polishing material in powdered form and as a pumice stone^21^. Different agents have been used to modify adsorbents to improve their adsorption capacity; various pumice modifications were tested in previous studies. Pumice modification effectively removes phosphate ions from water^22^. Iron-coated pumice was a promising adsorbent in removing NOM from water^23^. Magnesium chloride and hydrogen peroxide were used to modify the surface of natural pumice to increase the adsorbent's specific surface to remove fluoride^24^. Pumice modification by acid increased the adsorbent efficiency in humic acid removal from water^25^.

This study's major goal was to use a low-cost adsorbent for water purification using a locally plentiful adsorbent. Even though there has been earlier research on the removal of dye compounds using pumice stone, the present study stands out because it uses nanosized pumice to examine how the increased surface area of the pumice affects the adsorption process. Therefore, the use of a modified nano-scaled pumice as a novel adsorbent for dye compound treatment from water was studied in the present research. Response surface methodology (RSM) with central composite design (CCD) was also employed to ascertain the relationship between the effectiveness of dye removal and certain factors and optimize the adsorption process.

## Materials and method

The use of HMNP as an adsorbent for removing AR18 from aqueous solutions was investigated. All tests were conducted on a lab scale, and the effect of different parameters such as pH, adsorbent dose, contact time and initial dye concentration were examined.

### Material and reagents

Alvan Sabet Company, Hamedan, Iran, provided AR18 dye. All other chemicals like hexadecyl trimethyl ammonium chloride, Sodium hydroxide (NaOH, 1N) and hydrochloric acid (HCl, 1N) were purchased from Merck (Darmstadt, Germany).

### Batch adsorption studies

Experiments were carried out with 25 ml of dye solution at different concentrations. pH adjustment was done using NaOH and HCl 0.1 N. Samples were contacted with a desirable amount of adsorbent (0.5–3 g/l) on a shaker with 200 rpm at room temperature and a certain contact time. After adsorption, samples were centrifuged at 4500 rpm for 10 min. The studied variables in the present research were pH (3–9), adsorbent dosage (0.5–3 g/l), contact time (10–90 min) and initial dye concentration (10–70 mg/l). The amount of adsorbed dye was calculated by using the mass balance Eq. (1):

1 $${\mathrm{q}}_{\mathrm{e}}=\left({\mathrm{C}}_{0}-{\mathrm{C}}_{\mathrm{e}}\right)\frac{\mathrm{v}}{\mathrm{m}}$$

where C_0_ and Ce are the initial and final concentration of dye (mg/l), M represents the mass of adsorbents (g), and V is the volume of AR18 solution (L).

To determine the pH point of zero charge (pH_ZPC_) for HMNP, 0.2 g/L of the adsorbent in 30 ml of NaCl solution (0.01M) with different initial pHs (2–4–6–8–10–12) was shaken for 24 h. then the final pH of the solutions was measured and plotted versus initial pHs. The pH in which the curve crosses the line (final pH = initial pH) is taken as the pHzpc^26^.

### Analytical measurements

**Figure 1:**
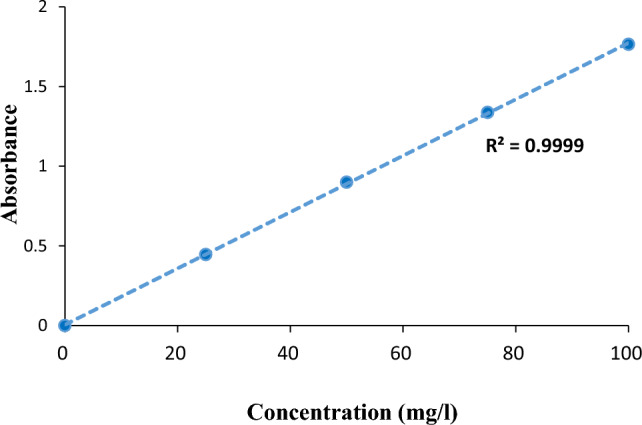
AR18 Calibration Curve.

To obtain demanded concentration of AR18 (10–70), the first stock solution was prepared by dissolving 0.5 of dye in 1 L distilled water and other desirable concentrations were prepared from the stock solution. Concentrations of dye solution were read at λ = 507 (nm) using a UV–visible spectrophotometer (Perkin Elmer Lambda 25) (Fig. 1).

### Adsorbent preparation

The pumice stone came from Iran's province of Azerbaijan. In the mineral processing lab at the mineral processing laboratory, school of mining engineering college, campus university of Tehran, Tehran, Iran, the raw pumice stone was crushed using a rad mill to a size of 100 mm. After that, a planetary ball mill transformed powdered pumice into Nano-pumice. At the central lab, Amirkabir University of Technology, Tehran, Iran. Nano-pumice was first thoroughly cleaned with distilled water; after that, the surface porosity was increased by contacting the adsorbent with a 1N HCL solution for 48 h at room temperature. The adsorbent was then fully washed with distilled water to achieve effluent turbidity of less than 1 NTU and a pH of 7, and the pumice was then dried in an oven at 105 °C for eight hours.

### Adsorbent modification

Nano-pumice was modified with a cationic surfactant hexadecyl-trimethyl ammonium-chloride (HDTMA. Cl) (2.5 mmol) solution. pH was adjusted to 10 by adding NaOH 1N, then shaken for 10 h. at room temperature at 220 rpm. After this time, filtered and dried at 120 °C for 2.5 h., then washed with distilled water and again dried at 120 °C for 6 hr^27^.

### Adsorbent characterization

Field Emission Scanning Electron Microscope (FESEM) was used to assess the surface morphology of Nano-pumice. TEM photograph was applied to determine the particle size. The specific area and mean pore diameter were determined by BET, XRD, XRF, and FTIR were also used for more adsorbent analysis.

### Adsorption Isotherms and kinetics

Isotherm studies were performed under optimum conditions with different dye concentrations (25–70mg); the added adsorbent to each sample was 2.375 g/l with solution pH = 4.2, and then samples were shaken at 200 rpm for 70 min. Kinetic studies with optimized parameters were conducted at contact time in the 10–70 min range. To determine dye adsorption on HMNP, three kinetics models and four isotherm models were examined.

### Adsorption Thermodynamics

The Thermodynamic of AR18 adsorption on HMNP was evaluated at optimum conditions (pH = 4.5, adsorbent dose = 2.375 g/l, contact time = 70 min, initial AR18 concentration = 25 mg/l) and different temperatures (15–55 °C) to find the performance of adsorption process. Thermodynamics parameters of adsorption were assessed by Gibbs free energy changes (ΔG^0^), enthalpy changes (ΔH^0^) and entropy changes (ΔS^0^) and sticking probability (SP*)[Eqs. 2–5]:

2 $$ \Delta {\text{G}^\circ} = - {\text{RT lnK}} $$

3 $$ \Delta {\text{G}^\circ} = \Delta {\text{H}^\circ} - {\text{T}}\Delta {\text{S}} $$

4 $$\mathrm{lnK}=-\frac{\mathrm{\Delta H}^\circ }{\mathrm{RT}}+\frac{\mathrm{\Delta S}^\circ }{\mathrm{R}}$$

5 $$ {\text{SP}} * = \left( {{1} - \beta } \right){\text{exp}} - \left( {{\text{Ea}}/{\text{RT}}} \right) $$

where Ea. is the activation energy (kJ/mol), T is the temperature (K), K is the sorption equilibrium constant, and β is surface coverage.

### Experimental design

Experiments were designed using Design-Expert11 (Stat. Ease.Inc Minneapolis, USA) software with Response Surface Methodology (RSM). A central composite design was applied to evaluate the effect of 4 different variables on the adsorption process (pH, initial concentration, contact time, and adsorbent dosage). CCD requires centre points, axial points, and cube points. The total number of experiments can be determined by presented Eq.(6)

6 $$ {\text{N}} = {\text{2k}} + {\text{2k}} + {\text{C}}_{0} $$

K represents the number of experimental variables, 2k is the cubic runs, 2k is the axial runs, and C_0_ is the centre point's runs. Table 1 presents independent variables and the levels of each variable, while CCD and coded factor values are shown in Table 2.

**Table 1:** Independent variables and the levels of each variable.

Variable	Symbol	− α	− 1	0	+ 1	+ α
pH	X_1_	3	4.5	6	7.5	9
Adsorbent dosage (g/L)	X_2_	0.5	1.125	1.75	2.375	3
Contact time (min)	X_3_	10	30	50	70	90
Acid red 18 concentration (mg/L)	X_4_	10	25	40	55	70

**Table 2:** CCD Design for AR18 Removal.

Std	Run	A: pH	B: Dose	C: Time	D: AR18	Ce	RE
			g/L	Min	mg/l	mg/l	%
20	1	6	3	50	40	7.46	81.34
1	2	4.5	1.125	30	25	9.91	60.34
3	3	4.5	2.375	30	25	1.40	94.38
30	4	6	1.75	50	40	23.2	42
13	5	4.5	1.125	70	55	36.85	33
29	6	6	1.75	50	40	22.61	43.46
10	7	7.5	1.125	30	55	50.05	9
17	8	3	1.75	50	40	11.26	71.85
26	9	6	1.75	50	40	22.93	42.66
5	10	4.5	1.125	70	25	6.5	74
18	11	9	1.75	50	40	27.46	31.41
25	12	6	1.75	50	40	24.32	39.18
2	13	7.5	1.125	30	25	14.79	40.84
19	14	6	0.5	50	40	33.34	16.65
23	15	6	1.75	50	10	0.2	98
6	16	7.5	1.125	70	25	13.84	44.64
16	17	7.5	2.375	70	55	33.22	39.6
27	18	6	1.75	50	40	21.15	47.11
22	19	6	1.75	90	40	20	50
12	20	7.5	2.375	30	55	29.87	45.69
9	21	4.5	1.125	30	55	43.43	21.03
21	22	6	1.75	10	40	21.9	45.25
4	23	7.5	2.375	30	25	3.81	84.76
24	24	6	1.75	50	70	50.4	28
7	25	4.5	2.375	70	25	0.25	99
28	26	6	1.75	50	40	23.78	40.55
11	27	4.5	2.375	30	55	27.81	49.43
14	28	7.5	1.125	70	55	48.97	11
15	29	4.5	2.375	70	55	22.99	58.2
8	30	7.5	2.375	70	25	4.78	80.88

## Results and discussion

### XRD

**Figure 2:**
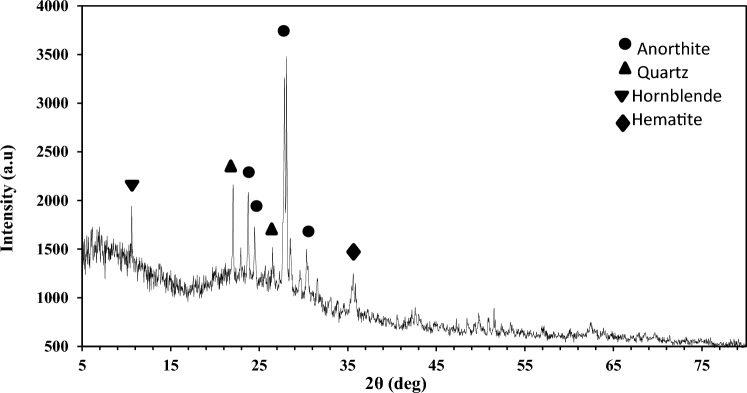
XRD of Nano-pumice.

X-ray diffraction of pumice was analyzed in the range of 2θ = 5–80° and step size = 0.02; the result is shown in Fig. 2. Major constituents of Nano-pumice are Anorthite (CaAl_2_Si_2_O_8_), Quartz (SiO_2_), Hematite (Fe_2_O_3_) and Hornblende (Ca, Na)_2_(Mg, Fe, Al)_5_(Al, Si)_8_O_22_(OH)_2_). The main peaks were observed at 2θ = 10.60°, 22.06°, 23.78°, 24.48°, 26.51°, 28.10°, 30.33° and 35.64°^28–30^. A dome at 2θ = 20–40° can confirm the more amorphous phase of Nano-pumice^31^. The presence of Quartz in this analysis shows good agreement with the high percentage of SiO_2_ in the sorbent, as reported in Table 3.

**Table 3:** Nano-pumice XRF.

Compound	Percent%
SiO_2_	68.79
Al_2_O_3_	8.82
Fe_2_O_3_	6.34
CaO	6.42
SO_3_	2.99
K_2_O	2.29
Na_2_O	1.2
MgO	0.88
TiO_2_	0.68

### XRF studies

X-ray Fluorescence Analysis was done to determine the chemical composition of pumice (Oxford Instruments, ED2000). The results are given in Table 3. As can be seen from the results, SiO_2_ and Al_2_O_3_ are the two main compounds in the Nano-pumice sample.

### FESEM and TEM

**Figure 3:**
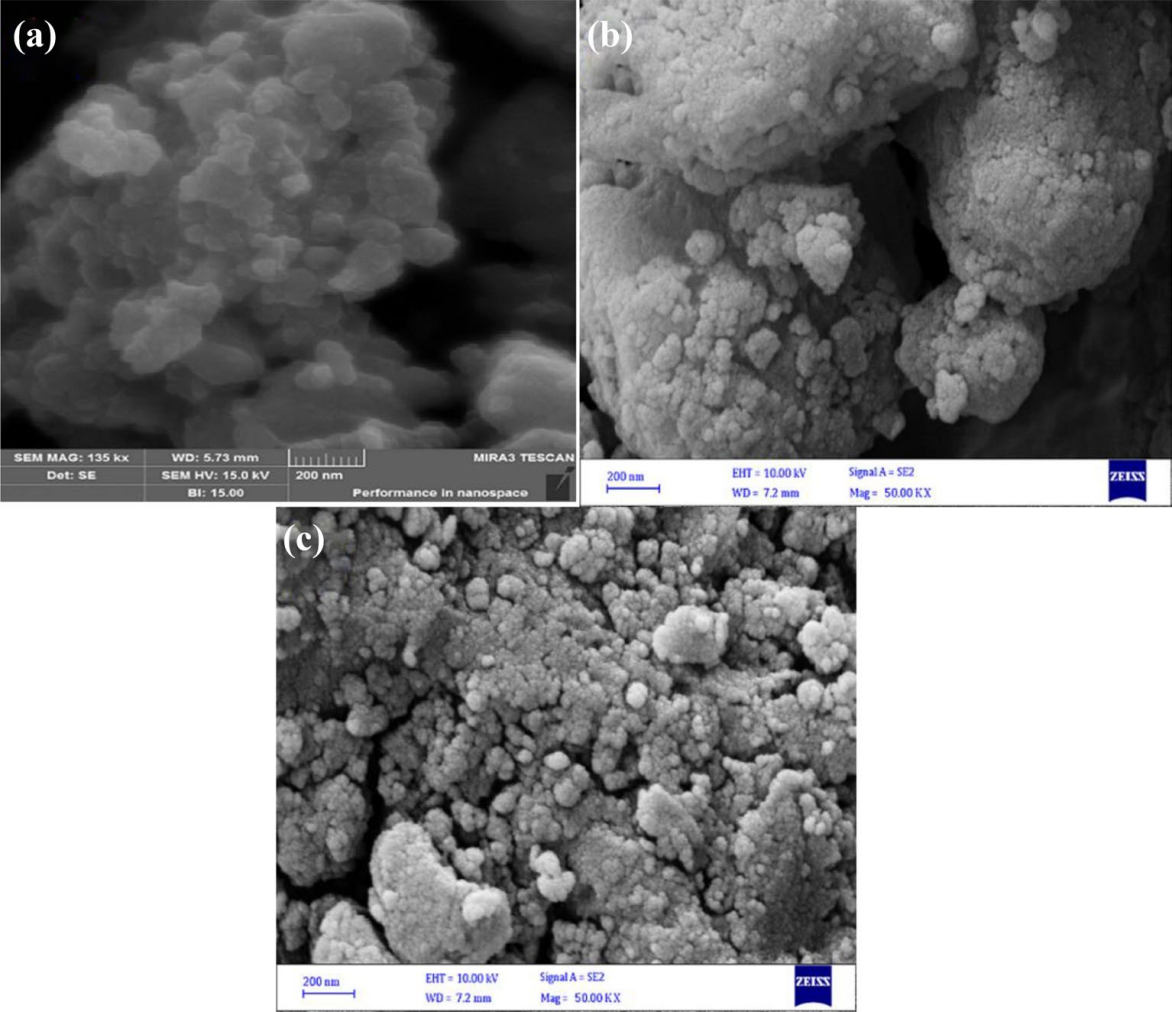
FESEM images of Nano-pumice [(**a**) (NP), (**b**) (HMNP), (**c**) (after adsorption HMNP)].

**Figure 4:**
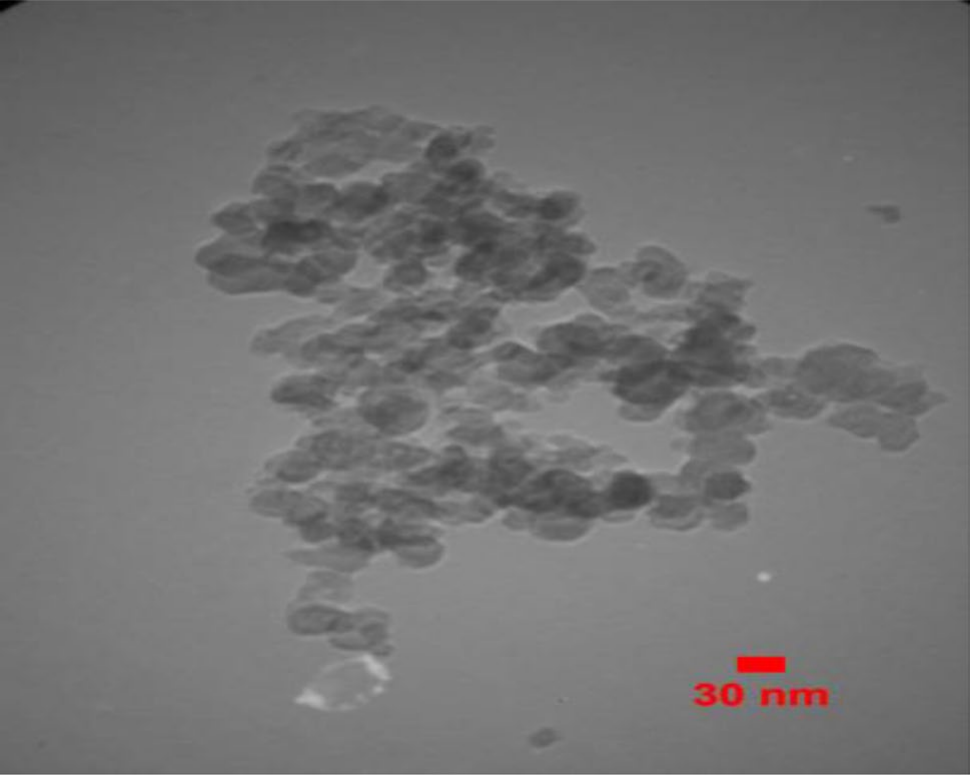
TEM of Nano-pumice.

While HMNP has become more agglomerated with a smooth surface and is extremely porous, showing more accessible sites for dye adsorption (Fig. 3b), NP exhibits sharp edges and a rough surface texture in FESEM images (Fig. 3a). The image taken following the adsorption procedure demonstrates how dye molecules filled the pores and surfaces of the HMNP. (Fig. 3c). Particle size can be understood from the TEM image of nano pumice in Fig. 4, and it is also clear that the particles are agglomerated and have a semi-polygonal form.

#### EDAX analysis

**Figure 5:**
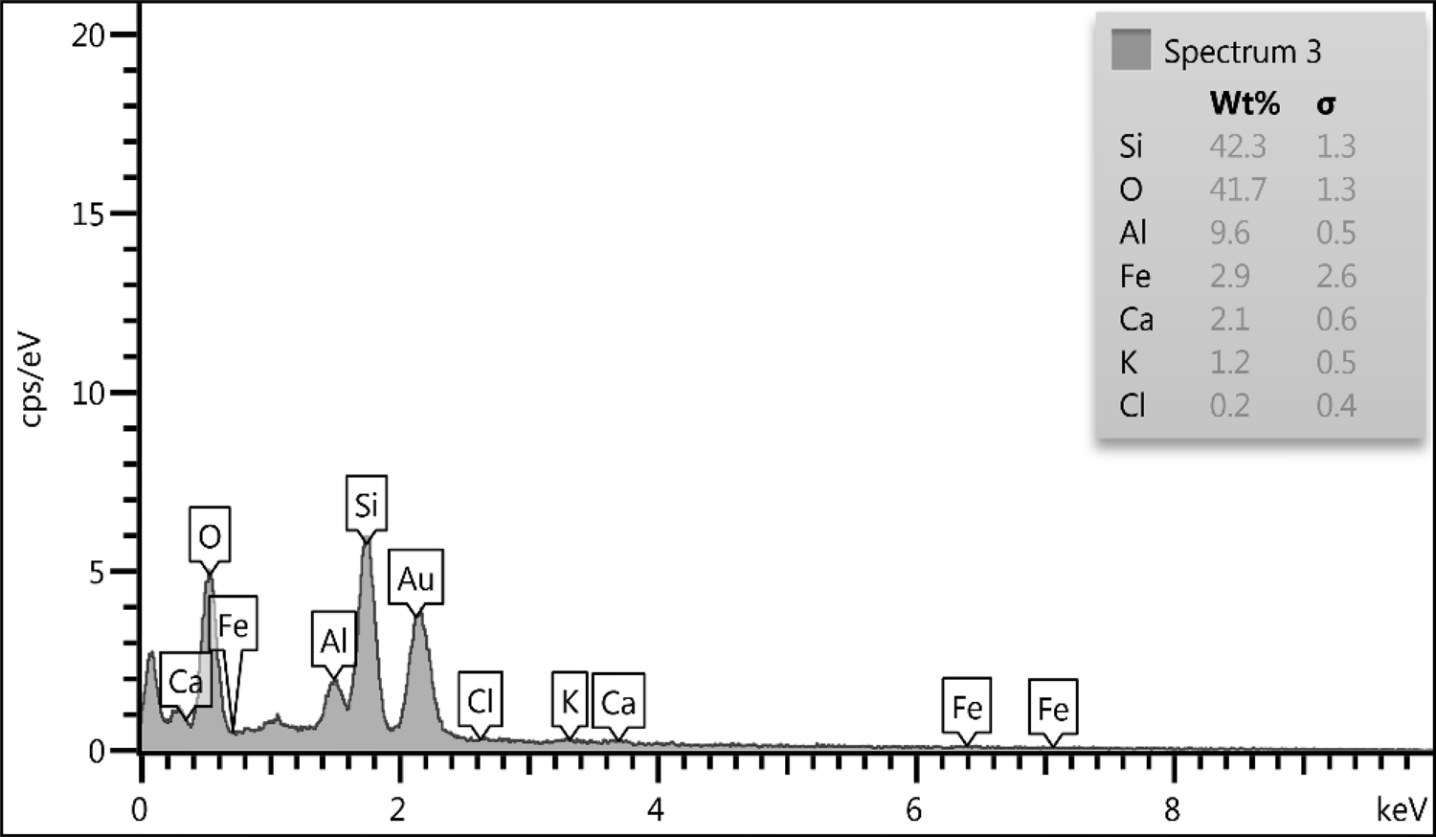
Energy-dispersive X-ray spectroscopy of HMNP.

Elemental constituent of HMNP material determined by Energy-dispersive X-ray spectroscopy (EDX). From the results in Fig. 5, it is obvious that the major contents are Si and oxygen, with 42.3% and 41.7%, respectively; other elements are Al, Fe, Ca, K, and Cl.

### BET

**Figure 6:**
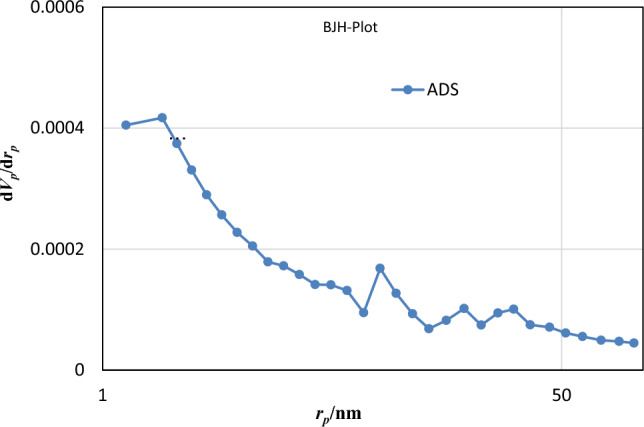
HMNP pore size distribution.

BET (Microtrac Bel Corp, BElSORP Mini) analysis (N_2_ gas adsorption method) was used to calculate total pore volume, specific surface area and average pore diameter of NP and HMNP. The results of BET are shown in Table 4. The increased surface area by modification of Nano-pumice (from 1.49 to 10.27) is aligned with previous studies^32,33^. The pore size distribution calculated by the BJH method is displayed in Fig. 6. As it can be seen, HMNP pore size distribution is between 1 and 100 nm, and most of the particle's pore sizes are 2–50 nm which shows the mesoporous size of the sorbent.

**Table 4:** BET analyzes pore volume and average pore diameter of Nano-pumice.

Sorbent	Specific surface area (m^2^ g^−1^)	Average pore diameter (nm)	Average pore volume (cm^3^/g)
Natural Nano-pumice	1.4921	26.015	0.066821
HMNP	10.274	20.233	0.0075472

### FTIR Analysis

**Figure 7:**
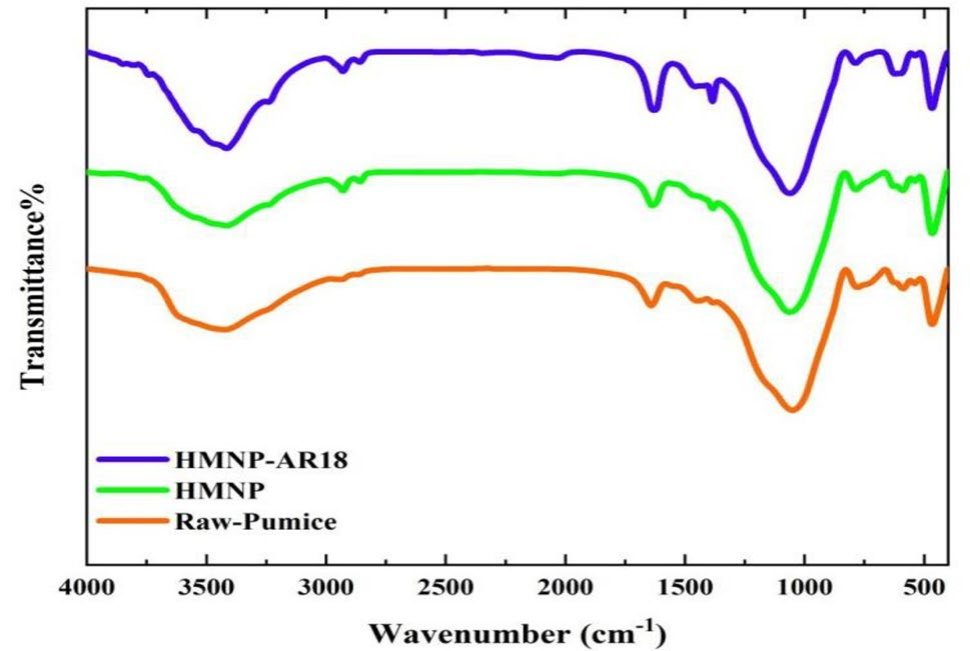
FTIR of Nano-pumice.

To obtain the functional groups of the pumice sample, the Fourier transform infrared analysis was performed (PerkinElmer, Spectrum Two) while the analysis range was between 400 and 4000 cm^−1^. Figure 7 shows the FTIR spectra of NP, HMNP, and after adsorption, HMNP Peaks at 3415–3423 cm^−1^ were related to water molecules ^18^. 1049 cm^−1^ and 1060 cm^−1^ have appeared in NP, HMNP and after adsorption HMNP, which are related to Si–O and Si–Al stretching vibration ^34^. 1625–1641 cm^-1^ showing stretch vibration of band O–H. Si–O–Al band was located at 779–786 cm^-1^ while near 466 cm^-1^ bending vibration of the Si–O–Si band was identified^23^. The band around 587–621 cm^-1^ is associated with the bending vibration of Fe–O3^20^. In HMNP and after adsorption HMNP, two new peaks were observed at 2928 and 1384 cm^-1^ related to C–H band and C=O, respectively^35,36^. The peak at 2033 cm^-1^ found after adsorption of HMNP may be due to the C–O bond.

### Statistical analysis

The validity of linear, 2FI, Quadratic and Cubic models was assessed. The Quadratic model was selected with an insignificant lack of fit (0.6656), confirming that the model is valid with Adjusted R^2^ = 0.9898 and Predicted R^2^ = 0.9787. Data for all fitted models are shown in Table 5. As can be seen from Table 6, different variables have their effects on dye adsorption. In this study, initial dye concentration showed the most impact on dye adsorption efficiency with an F- value of 1354.54.

**Table 5:** Fitted models data for AR18 removal.

Source	Sequential p-value	Lack of Fit P-value	Adjusted R^2^	Predicted R^2^	
Linear	< 0.0001	0.0162	0.9233	0.9090	
2FI	0.4546	0.0145	0.9233	0.9122	
Quadratic	< 0.0001	0.6656	0.9898	0.9787	Suggested
Cubic	0.4114	0.8526	0.9908	0.9773	Aliased

**Table 6:** ANOVA for Quadratic model.

Source	Sum of squares	df	Mean square	F-value	p-value	
Model	17,843.65	14	1274.55	202.91	< 0.0001	Significant
A-pH	1905.49	1	1905.49	303.35	< 0.0001	
B-Dose	6255.54	1	6255.54	995.87	< 0.0001	
C-Time	81.96	1	81.96	13.05	0.0026	
D-Dye	8508.52	1	8508.52	1354.54	< 0.0001	
AB	67.28	1	67.28	10.71	0.0051	
AC	116.59	1	116.59	18.56	0.0006	
AD	25.58	1	25.58	4.07	0.0619	
BC	49.04	1	49.04	7.81	0.0136	
BD	25.78	1	25.78	4.10	0.0609	
CD	0.1502	1	0.1502	0.0239	0.8792	
A^2^	144.14	1	144.14	22.95	0.0002	
B^2^	73.20	1	73.20	11.65	0.0038	
C^2^	45.73	1	45.73	7.28	0.0165	
D^2^	723.21	1	723.21	115.13	< 0.0001	
Residual	94.22	15	6.28			
Lack of Fit	56.95	10	5.69	0.7639	0.6656	Not significant
Pure error	37.27	5	7.45			
Cor total	17,937.88	29				

On the other hand, time had the lowest effect. Also, the interaction between A and C significantly affects adsorption due to its bigger F value among all other interaction variables. The quadratic function of D also showed the highest effect on dye adsorption compared with three others (A^2^, B^2^, and C).

**Figure 8:**
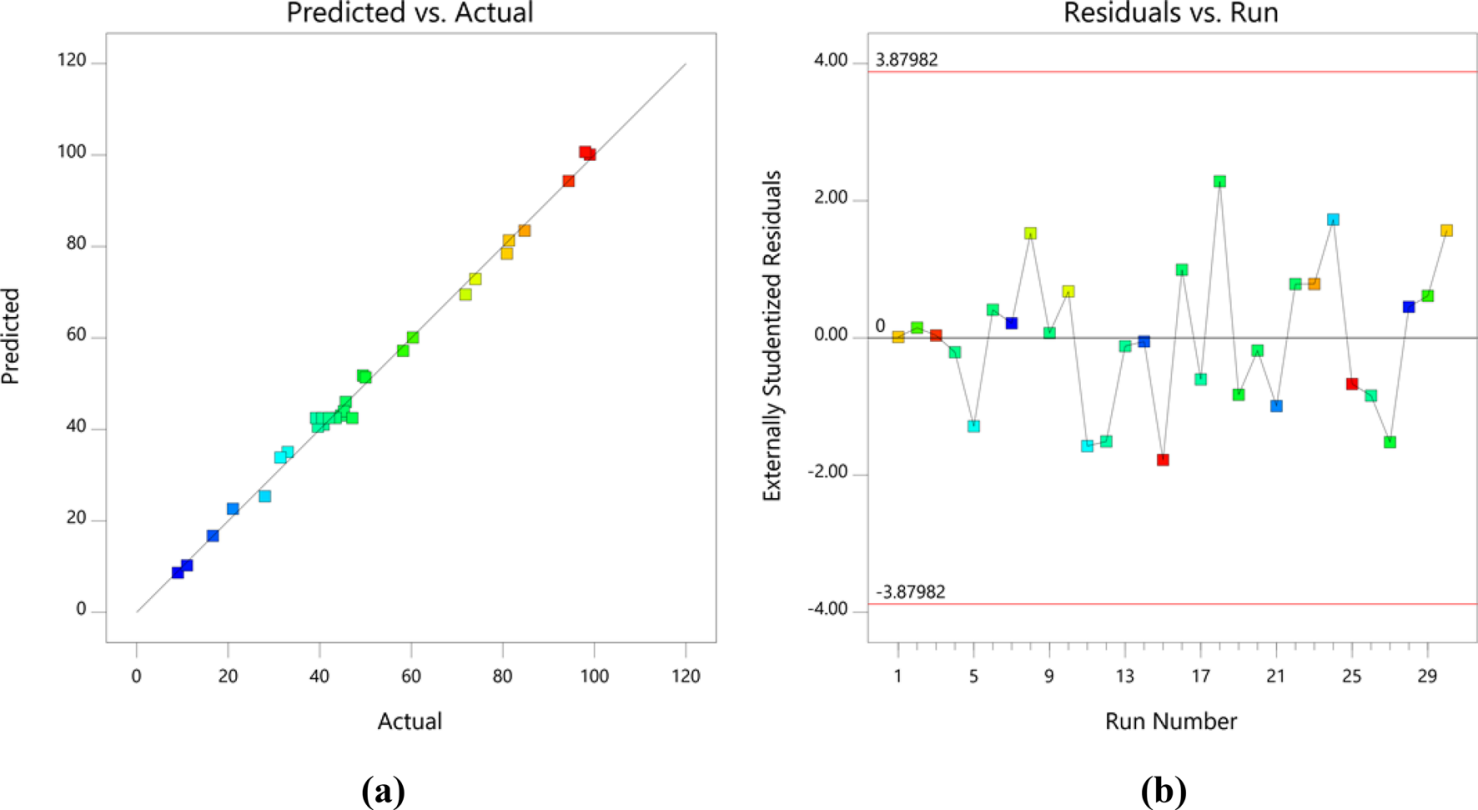
Predicted vs. actual value of AR18 removal (**a**) residual vs. the run (**b**).

Figure 8a displays predicted vs. actual efficiency in AR18 removal from solution by HMNP, which shows a good correlation between obtained experimental efficiency and predicted efficiency by software. In Fig. 8b, the residual amount of each run is shown, indicating a small difference between them (the highest and the lowest amounts were between 2 and  − 2).

### Interactive effect of pH and adsorbent dosage

**Figure 9:**
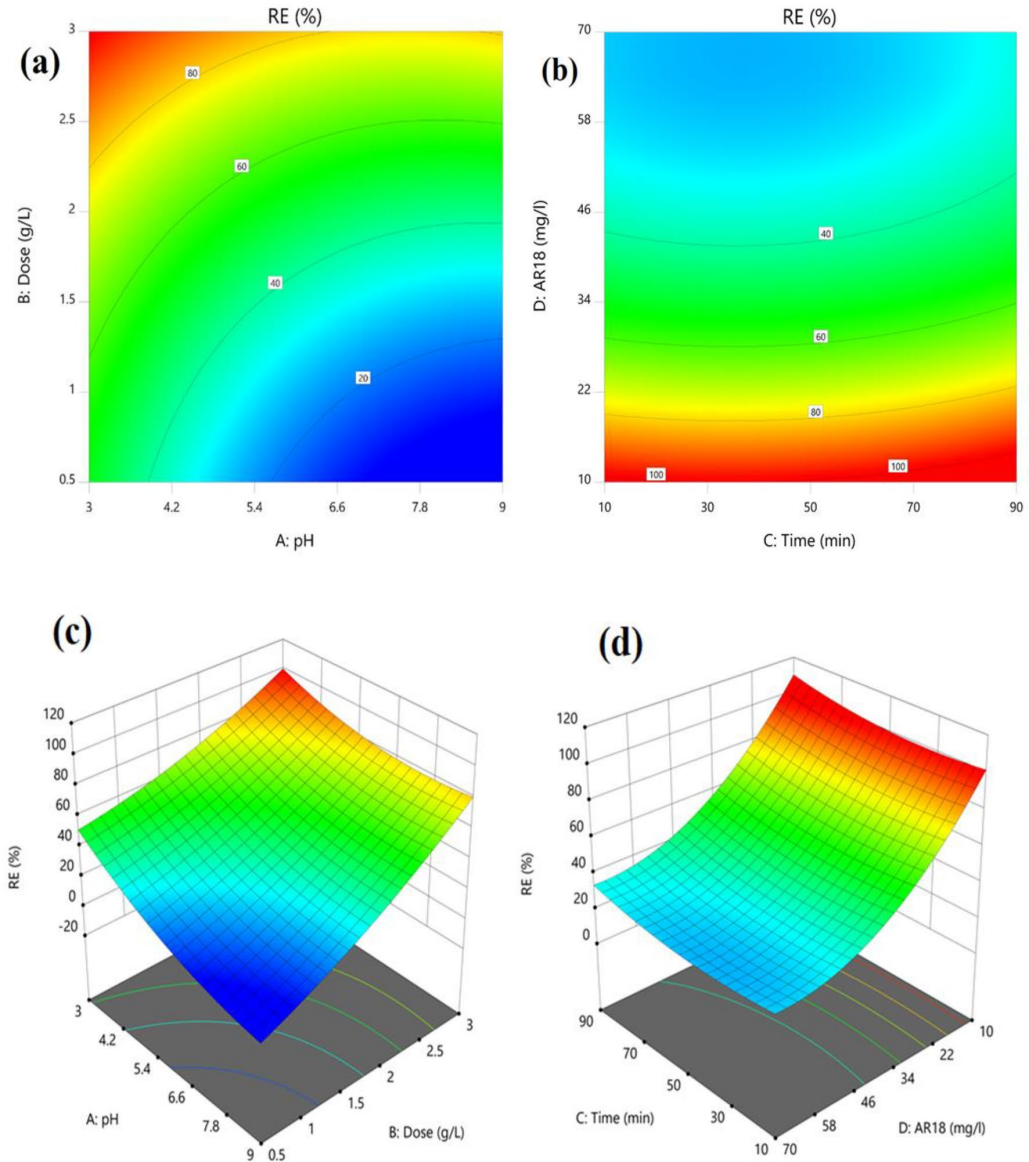
2D and 3D and contour plots showing the effect of pH and adsorbent dose (**a**, **c**), initial concentration of AR18 and time (**b**, **d**).

According to Fig. 9a,c and the negative coefficient of pH, dye removal effectiveness declines as pH rises. AR18 adsorption increases dramatically at pH < 4.2 but gradually at pH > 4.2. The existence of more surface positive charges on the adsorbent at lower pHs and negative charges on the dye molecules, and the resultant electrostatic sorption between them, can be used to explain why AR18 removal is higher at acidic pHs^37^. The calculated pH_ZPC_ value for HMNP was 5.6. It implies that the sorbent's surface is positively charged when the pH of the solution is lower than pH_ZPC,_ and adsorbent surfaces become negatively charged at pH levels above pH_ZPC_ value, which causes dye ions to repel one another and reduce AR18 adsorption. Whereas at pH = 5.6, surface charges are zero^38^. As seen from Fig. 9a,c, increasing the adsorbent dosage increased the effectiveness of dye removal. On the other hand, adding more HMNP (0.5–3 g/l) increased the adsorption efficiency. It is most likely because more sites for dye adsorption can be provided with higher dosages. This outcome is consistent with earlier research^39^.

### Interactive effect of time and initial concentration

In Fig. 9b, d, The removal efficiency increases from 40 to about 100% as the initial concentration of AR18 decreases, particularly from 45 to 10 mg/l. It also increases when the sorbent has more time to contact the dye molecules, from 10 to 90 min. In the time range of 10–90 min, the impact of contact time was examined. It was discovered that as the contact time grew, more dye was absorbed. It makes sense that as the amount of time increases, more dye molecules have a chance to adsorb on the surface of the HMNP. This result is in agreement with other studies^40^. Initial dye concentration is the primary factor affecting the effectiveness of dye adsorption and significantly impacting the absorption rate. Initial dye concentration has a reverse effect on dye removal. While the initial concentration increases, dye removal decreases. A possible explanation is adsorbent's free sites are occupied when the concentration is high^41^.

### Development of regression model equation

CCD was used for the development of mathematical equations. The highest efficiency was 99%. The final equation shows the empirical relationship between dye removal (Y) based on pH (A), dosage (B), time (C) and initial dye concentration (D):

7 $$ {\text{Y}} = + {42}.{49} - {8}.{\text{91A}} + {16}.{\text{14B}} + {1}.{\text{85C}} - {18}.{\text{83D}} + {2}.0{\text{5AB}} - {2}.{7}0{\text{AC}} + {1}.{\text{26AD}} - {1}.{\text{75BC}} - {1}.{\text{27BD}} - 0.0{\text{969CD}} + {2}.{\text{29A}}^{2} + {1}.{\text{63B}}^{2} + {1}.{\text{29C}}^{2} + {5}.{\text{13D}}^{2} $$

Insignificant terms (p values > N 0.05) were dismissed for the Development of the regression model equation:

8 $$ {\text{Y}} = + {42}.{49} - {8}.{\text{91A}} + {16}.{\text{14B}} + {1}.{\text{85C}} - {18}.{\text{83D}} + {2}.0{\text{5AB}} - {2}.{7}0{\text{AC 1}}.{\text{75BC}} + {2}.{\text{29A}}^{2} + {1}.{\text{63B}}^{2} + {1}.{\text{29C}}^{2} + {5}.{\text{13D}}^{2} $$

#### Determining optimal settings

Using the numerical optimization method from software, the maximum efficiency (Eff = 99%) was determined to occur at pH = 4.5, adsorbent dose = 2.375 g/l, contact time = 70 min and initial AR18 concentration = 25 mg/l. But the best AR18 removal efficiency was achieved at 98.8% in practice with the mentioned conditions.

#### Isotherm studies

**Figure 10:**
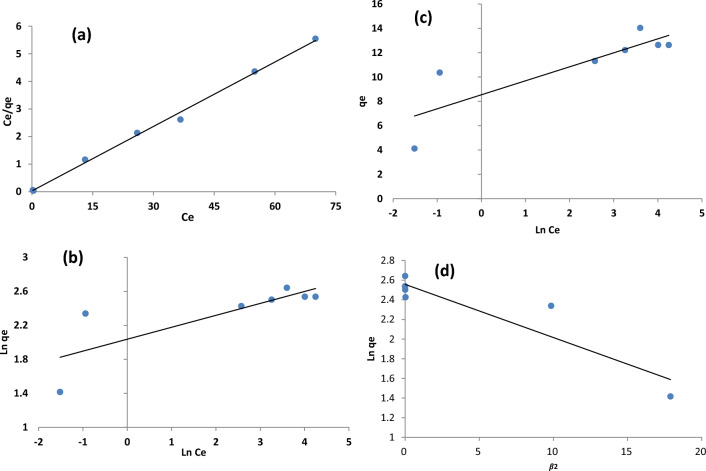
Isotherm models for AR18 adsorption: (**a**) Langmuir (**b**). Freundlich (**c**) Temkin (**d**) Dubinin-Radushkevich.

To realize the nature of the interaction between dye molecules and HMNP adsorption isotherms is necessary^42^. In the present study to model the relationship between adsorbed dye on the adsorbent and remained dye in solution, Langmuir, Freundlich, Temkin and Dubinin-Radushkevich models were used (Their plots are shown in Fig. 10), obtained parameters and constants are shown in Table 7. Isotherm models give a better understanding of the adsorption mechanism. To carry out isotherm’s studies, all parameters were at their optimized conditions with pH = 4.5, adsorbent dose = 2.375 g/l, contact time = 70 min and initial AR18 concentration in the range of 10–70 mg/l at room temperature. To corroborate the fitted model, the correlation coefficients were used.

**Table 7:** Isotherm and Kinetic models parameters and constants for AR18 removal by HMNP.

	Langmuir			Freundlich			Temkin			Dubinin-Radushkevich		
	R2	qmax(mg/g)	b	R2	Kf(L/mg)	nF	R2	bT	AT	R2	qmax(mg/g)	β
Isotherm	0.996	12.84	2.64	0.641	7.77	6.28	0.71	1.3	0.038	0.85	12.92	5.41*10–8

	Pseudo-first order			Pseudo-second order			Intraparticle diffusion					
	R^2^	qe (mg/g)	K1 (min^-1^)	R^2^	qe (mg/g)	k_2_(g/mg.min)	R^2^		C (mg/g)		K_dif_ (mg/g min^0.5^)	
Kinetic	0.968	3.279	0.047	0.999	10.834	0.025	0.96		7.311		0.378	

Langmuir, Freundlich, Temkin, and Dubinin-Radushkevich are expressed by following equations (Eqs. 7–10) respectively in Table 8. Langmuir model is valid for the monolayer adsorption of a liquid on a homogenous adsorbent surface^43^. Where q_max_ (mg/g) is the maximum adsorption capacity of the adsorbent, C_e_ is defined as equilibrium concentrations, and b is the adsorption rate constant related to the energy of adsorption (l/mg), the larger b value depicts a larger affinity of adsorbent to the pollutant^44^. From obtained data, the maximum sorption capacity and adsorption energy for HMNP were 12.84 mg/g and 2.64 l/mg, respectively. Additionally, the high b value (2.64) points out the strong binding of AR18 on the HMNP surface.

**Table 8:** Kinetic and isotherm models parameter.

Model	Equation	plot	Parameters	Eq. no	Ref
Isotherm					
Langmuir	q_e_ = qmbC_e_/1 + bC_e_	_Ce/qe vs. Ce_	$q_{m} \frac{1}{slope}$	(7)	^6^
			$$K_{L} = \frac{1}{{{\text{Int}}ercept \times q_{m} }}$$		
Freundlich	Log q_e_ = Log K_*f*_ + (1/n_F_) log C_e_	_log (qe) vs. log (Ce)_	$K_{f} = \exp ({\text{int}} ercept)$	(8)	^7^
			$$slope = \frac{1}{n}$$		
Temkin	q_e_ = RT/b_T_ ln(A_T._C_e_)	_ln Ce vs. qe_	*b*_*T*=_ *slope*	(9)	^7^
			*ln A*_*T*_ = *intercept/B*_*T*_		
Dubinin-Radushkevich	lnq_e_ = lnq_max_—βϵ^2^	Ln q_e_ vs. ϵ^2^	*K* = *slop*	(10)	^8^
			*q*_*max*_ = *intercept*		
Kinetic					
Pseudo-first-order	ln(q_e_ − q_t_) = − k_1_ t + ln(q_e_)	log(*q*_e_ − *q*_*t*_) vs. *t*	*k*_*1*_ = *slope*	(11)	^9^
			*q*_*e*_ = *intercept*		
			*1/q*_*e*_ = *Slope*		
Pseudo-second-order	t/q_*t*_ = 1/(k_2_q*e)* ^2^ + (t/q*e*)	t/q_t_ vs. t	*1/(k*_*2*_*q*_*e*_^*2*^*)* = *Intercept*	(12)	^9^
Intra-particle diffusion	q_t_ = K_dif_ t^1/2^ + C	t vs.q_t_	*C* = *Intercept*		
			*K*_*dif*_ = *Slope*	(13)	^9^

Freundlich model uses for adsorption on heterogeneous surfaces with the interaction between adsorbed molecules and describes heterogeneous systems^45^. K_F_ is the Freundlich constant (l/g) is the adsorption or distribution coefficient, and the 1/n_F_ value indicates the degree of non-linearity between solution concentration and the adsorption process^46^. Furthermore, 1/n_F_ > 1 demonstrates cooperative adsorption, while 1/n_F_ < 1 implies a normal Langmuir adsorption^47^. The result of experimental data from the Freundlich model showed 1/n_F_ > 1 (0.159), which reveals that the adsorption process of AR18 removal follows a normal L-type Langmuir adsorption. Besides, the coefficient 1/n (generally 0–1) indicates the favourable adsorption of the adsorbate to adsorbent^42^. Temkin isotherm model considers the effects of indirect adsorbent–adsorbate interaction on adsorption isotherms and heat of adsorption^42^. BT = (RT)/b_T_, T is the absolute temperature (Kelvin), R is the universal gas constant (8.314 J/mol K), b is the heat of adsorption constant, and A_T_ (L/g) is the binding constant^46^.

The D–R isotherm model is used to identify the nature of the adsorption process as physical. Where ϵ is Polanyi potential, β is a constant for the free energy of adsorption^48^. The affinity between AR18 and HMNP can be estimated by the R_L_ constant, which is dimensionless.

9 $$ {\text{RL}} = {1}/{1} + {\text{bC}}_{0} $$

b (L/mg) is the Langmuir constant and C_0_ (mg/L) is the AR18 concentration. The value of R_L_ shows the nature of adsorption as follows^49^:

0 < R_L_ < 1 favorable, R_L_ > 1 unfavorable, R_L_ = 1 linear, R_L_ = 0 irreversible.

The calculated R_L_ value is between 0.003 and 0.03. As all of these values are 0 < R_L_ < 1, it can be understood that pumice has favourable adsorption.

In this study, Langmuir had a greater R^2^ value than other models, and it was obtained at 0.9962; for D-R, this value was 0.85; for Temkin, it was 0.71, and for Freundlich, it was 0.64, which means that the adsorption isotherms are in good agreement with Langmuir model and AR18 sorption on HMNP is a monolayer. Table 7 displays isotherm model parameters and constants for AR18 removal by HMNP.

#### Kinetic studies

**Figure 11:**
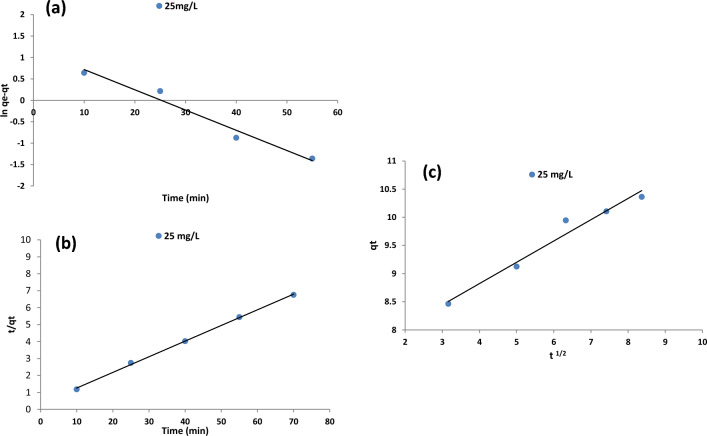
Kinetic Models for AR18 adsorption: (**a**). Pseudo-first order (**b**). Pseudo-second order (**c**) Intraparticle diffusion.

Kinetic studies were conducted to understand the adsorption mechanism and dye uptake rate. Pseudo-first-order, Pseudo-second-order, and Intra-particle diffusion models were used to analyze the adsorption kinetics and Eqs.11–13 present models, respectively (Table 8) in which q_e_ (mg/g) is the amount of adsorbed dye on the adsorbent at equilibrium conditions, q_t_ (mg/g) is the amount of adsorbed dye at any time. K_1_ (min^−1^), K_2_ (g/mg^.^min) and K_dif_ (mg/ g·min^0.5^) are the rate constants of pseudo-first-order, second-order and Intra-particle diffusion models, respectively. In this study, Pseudo-first-order describes the uptake rate based on adsorption capacity. The obtained data didn’t align with this model due to its low q_e_ compared to the calculated q_e_ and the low R^2^. The R^2^ value for the Pseudo-second order was obtained at 0.999, revealing that the adsorption process is best fitted into this model. Furthermore, the calculated qe value in the Pseudo-second-order model (qe cal = 10.834 mg/g) is closer to the experimental qe value (qe exp. = 10.36 mg/g). These results are in agreement with Gomez^50^ Kuczajowska-Zadrożna^51^ and Zhang^52^. The rate of adsorption site occupation is assumed to be proportional to the square of the number of empty sites by pseudo-second-order kinetic theory. Table 9 displays Kinetic model parameters and constants for AR18 removal by HMNP, and Fig. 11 represents kinetic model plots.

**Table 9:** Kinetic models parameters and constants for AR18 removal by HMNP.

Kinetic model	Parameters	Values
Pseudo-first order	R2	0.968
	qe (mg/g)	3.279
	K1 (min-1)	0.047
	R2	0.999
Pseudo-second order	qe (mg/g)	10.834
	k2(g/mg.min)	0.025
	R2	0.96
Intraparticle diffusion	C (mg/g)	7.311
	Kdif (mg/g.min0.5)	0.378

#### Thermodynamic studies

Temperature is an important parameter in the adsorption process. Thermodynamic studies were carried out at five different temperatures to understand the effect of temperature on dye removal efficiency. Table 10 indicates that increasing temperature increase k and q_e_, which suggests that AR18 removal by HMNP can have higher efficiency at a higher temperature. The obtained values of Gibbs free energy changes (ΔG^0^), enthalpy changes (ΔH ͦ) and entropy changes (ΔS ͦ ) are presented in Table 10. ΔH^0^ has a positive value (33.59 (kJ/mol)), meaning the adsorption process is endothermic. In other words, by increasing temperature, the removal efficiency increases since heating the active sites of adsorbents to high temperatures strengthens the bonds between the adsorbate molecules. Negative ΔG^0^ (between -1.978 and -6.938 kJ/mol) indicated the spontaneous nature of dye removal. The positive amount of ΔS° (0.117 kJ/mol) can ascertain the increased randomness at the solid/liquid interface^53^. These results are in agreement with previous studies^54^.

**Table 10:** The values of thermodynamic parameters of AR18 adsorption onto the HMNP.

T(^0^ K)	K	Ln K	q_e_ (mg/g)	ΔG^0^ (kJ/mol)	ΔH^o^ (kJ/mol)	ΔS^o^ (kJ/mol)
288	2.284	0.82634	8.888	− 1.97	33.59	0.117
298	4.949	1.59929	9.701	− 3.96		
308	7.802	2.054461	9.987	− 5.26		
318	8.4245	2.131155	10.025	− 5.63		
328	12.736	2.544499	10.189	− 6.93		

#### Adsorption mechanism

Ion exchange, physisorption, and chemisorption are the three main divisions of the adsorption mechanism. The term "physisorption mechanism" refers to surface adsorption that doesn't interfere with the adsorbent's electronic orbitals or the adsorbate. Van der Waals interactions, electrostatic interactions, hydrogen bonds, diffusion, and hydrophobic interactions could all be involved. The opposite scenario is the chemisorption mechanism which involves valence and electronic orbital forces between the absorbent and adsorbate. It produces an irreversible chemical connection to the adsorbent's surface. Complex formation, chelation, covalent bonding, redox reaction, and proton displacement can all be part of the mechanism behind the chemisorption process^53–55^. The ΔH^0^ value can be used to determine the physicochemical characteristics of adsorption; when it is between 0 and 20 kJ/mol, adsorption is physisorption; between 20 and 80 kJ/ mol, both physisorption and chemisorption occur; and between 80 and 400 kJ/mol, the adsorption is followed by chemisorption^56–58^. According to the calculated ΔH^0^ (Table 10), the adsorption type for HMNP is physical–chemical adsorption. Due to the positively charged surface of HMNP in low-pH solutions, AR18 removal increases. The opposing charges on the molecules of AR18 and AR18 bring about electrostatic attraction between HMNP and dye. However, the outcome indicates that the AR18 and HMNP have no attraction for one other at high pH. As a result, at high pH, the elimination of AR18 molecules is reduced. The findings thus imply that chemisorption may be the mechanism of AR18 elimination in a low-pH solution. The elimination process may involve physisorption at high pH levels. Alternatively, surfactants can be utilized to improve the adsorption capacity of mineral adsorbents. HDTMA is one of the most often utilized surfactants for modification. The interaction of mineral absorbents with the hydrophobic tails of HDTMA ions, which replaces the Na^+^ cation on the surface of the absorbent and causes the adsorbent’s surfaces to be positively charged, causes the increase in adsorption capabilities. Because of the electrostatic interaction between the adsorbate and the surfactant-modified adsorbent, anionic dyes could be adsorbed^59,60^.

#### Reusability study of HMNP

**Figure 12:**
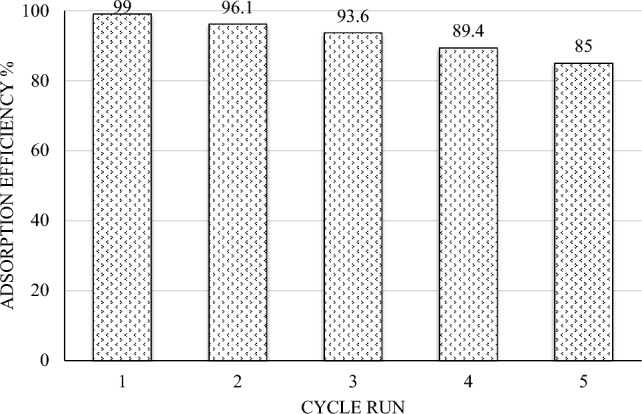
Reusability of HMNP for ten successive cycles (condition: pH = 4.5, sorbent dosage = 2.375 g/l, AR18 concentration = 25 mg/l, and contact time = 70 min).

For economic reasons, the reusability of the chosen adsorbent plays an important role in studies. By tracking the adsorption capacity of the adsorbent for five cycles under ideal conditions, the reusability of HMNP was examined. Desorption was carried out by eluting the AR18 adsorbed on HMNP with a 0.5 M NaOH solution following each run of adsorption. The modified pumices’ good recyclability for AR18 adsorption is demonstrated in Fig. 12, which depicts a reduction in HMNP’s adsorption effectiveness from 99 to 85% after five consecutive recycles. The reusability test results revealed that the prepared HMNP sorbent shows no considerable loss in its efficiency even after five cycles. Some previous studies on different contaminants like Antimony and phosphate prove this ^61,62^.

#### Comparison of adsorbent with other reported adsorbents

The adsorption capacity of this study was compared with other pumice adsorbents reported by other researchers. Other studies have investigated the removal of different pollutants by pumice, and their maximum adsorption capacities (Q_max_) are listed in Table 11. In most studies, the maximum adsorption capacity has occurred at acidic.

**Table 11:** Comparison of maximum adsorption capacity of pumice in adsorbing various contaminants.

	Adsorbent	Pollutant	pH	Isotherm	Kinetic	q_m_ (mg g^-1^)	Ref
1	Modified pumice with HCL and NaOH	Fluoride	6	Langmuir	PSO	65.5	^63^
2	Pumice	Cu(II)	5.31	–	–	1.43	^64^
3	Cationic Surfactant treated nano pumice	Humic acid	3	Langmuir	PSO	27.34	^65^
4	HNO_3_ modified pumice	Humic acid	3	Freundlich	PSO	23.4	^20^
5	HMNP	AR18	4.5	Langmuir	PSO	12.84	This study

## Conclusion

In the present study, HMNP adsorbent has been synthesized for the adsorption of dye-containing industrial wastewater. CCD did prediction and optimization of the AR18 removal process with RSM. From obtained ANOVA results, it was understood that the AR18 initial concentration has the highest effect on the adsorption process while contact time has the lowest. The maximum adsorption capacity of HMNP was 12.84 mg/g with C_0_ = 25 mg/l, adsorbent dosage 2.375 g/l and pH = 4.5. The Langmuir isotherm equilibrium model was found best fitted in this study, and adsorption kinetic data showed a good agreement with the pseudo-second-order. The adsorption process is defined to be endothermic and random due to the Positive ΔH^o^ and ΔS ^o^ values. Moreover, negative ΔG can be considered evidence for the spontaneous nature of HMNP. In comparison to other adsorbents, HMNP has a low maximum adsorption capacity. Due to its easy accessibility, abundance, non-toxicity, and eco-friendliness, HMNP can be considered a useful adsorbent for low concentrations of AR18 despite its low adsorption capability. Nonetheless, it might be able to adsorb other pollutants more effectively.

## Data Availability

All data generated or analyzed during this study are available from the corresponding author upon reasonable request.
